# Mediating Factors between Childhood Traumatic Experiences and Eating Disorders Development: A Systematic Review

**DOI:** 10.3390/children8020114

**Published:** 2021-02-06

**Authors:** María F. Rabito-Alcón, José I. Baile, Johan Vanderlinden

**Affiliations:** 1 Department of Psychology, Open University of Madrid (UDIMA), 28400 Madrid, Spain; joseignacio.baile@udima.es; 2 Campus Kortenberg, Universitair Psychiatrisch (Centrum KU Leuven), 3070 Kortenberg, Belgium; johan.vanderlinden@upckuleuven.be

**Keywords:** eating disorders, childhood trauma, mediating factors

## Abstract

Background: Many people with eating disorders often report having suffered some kind of childhood trauma. For this reason, many studies have attempted to explore the mediating factors between traumatic experiences and the development of eating disorders. The aim of our study is to conduct a systematic review of published works on the mediating factors between childhood trauma and the development of eating disorders. Method: This review was carried out up to 5 December, 2020, using the databases PsycInfo and PubMed, combining the keywords, and applying a set of inclusion and exclusion criteria. Results: A total of 18 articles were retrieved. After the articles were analyzed, a set of mediating factors between childhood trauma and the development of eating disorders was established, including pathological dissociation, difficulty with emotion self-regulation, body dissatisfaction, negative affect/depression, anxiety, general distress, self-criticism, and alexithymia, among others. Conclusions: In addition to evaluating trauma in eating disorders, these results highlight the importance of paying special attention to the presence of various possible mediating factors, which must be taken into account in the planning of therapeutic treatment. Identifying symptoms of trauma or eating disorders early on could prevent onset of more severe psychopathology during adulthood.

## 1. Introduction

Individuals with eating disorders (EDs) often report having suffered traumatic experiences during childhood [1,2]. EDs are serious psychiatric disorders, which alter cognitive function, judgment, emotional stability, and restrict the life activities of sufferers [3]. Studies carried out to explore these variables have shown this relationship to exist between EDs and traumatic experiences in childhood [3,4,5,6,7]. Child traumatic experiences are defined as all kinds of traumatic experiences occurring in childhood, which include emotional abuse, physical abuse, sexual abuse, emotional or physical neglect, rape, bullying by peers, witnessing domestic violence, and serious accidents that threatened the lives of subjects [5]. Having established this relationship, successive studies have been carried out to explore which variables might exist between having suffered traumatic childhood experiences and the later development of EDs in adolescence or adulthood.

To better understand the possible relationship between childhood traumatic experiences and EDs, some authors have considered the possible mediating role of various factors. Among these factors are mentioned dissociation [8], difficulties in regulating emotions [9], self-criticism (which involves constant and harsh self-scrutiny and chronic concerns about others’ criticism and may eventually be expressed in a variety of dysfunctional attitudes and behaviors) [10,11], and body dissatisfaction [8].

For one, elevated levels of dissociation have been reported in people with EDs [7,12]. Dissociation is understood as a psychological defense mechanism by which a person can escape the distress caused by stimuli and emotions related to unresolved childhood traumatic experiences [12,13].

In other studies, difficulties with regulating emotions have been linked to the development of EDs and the presence of traumatic experiences during childhood [9].

In this regard, another variable that has been studied is the presence of body dissatisfaction in patients with EDs who report childhood trauma. In these cases, various authors [8,14] hypothesize the possibility that the distress is not related to a drive for thinness but rather the desire to be unattractive or to punish one’s own body out of guilt for what had happened, as in the case of sexual abuse. At the same time, several authors also report difficulties in perceiving internal sensations in ED patients who have suffered childhood traumatic experiences. When the body is associated with traumatic experiences, these patients often experience feelings of disgust towards one’s own body [14].

However, the data is disputable due to the fact that the same type of childhood traumatic experiences are not assessed in all the studies. There is also lack of consistency among the instruments used to measure the same variables in some cases and the heterogeneity of the different samples [15,16].

A wide variety of studies exist which explore, independently and/or separately, the mediating factors between childhood traumatic experiences and EDs. However, there have been no general reviews of all the various mediating factors that have been explored to date. As such, this study aims to perform a systematic review of the works published on the mediating factors between childhood traumatic experiences and the development of EDs, updating the data of previous reviews [10,17] and adding studies performed about several types of childhood traumatic experiences. This is an important area of study and one that needs specific attention in children’s health given the fact that identifying symptoms of childhood traumatic experiences or eating disorders early on could prevent onset of more severe psychopathology during adulthood.

## 2. Method

The systematic review was carried out following the Preferred Reporting Items for Systematic Reviews and Meta-Analyses (PRISMA) standard [18]. Inclusion criteria comprised studies retrieved in which the participants included patients diagnosed with eating disorders based on DSM-III (Diagnostic and Statistical Manual of Mental Disorders) [19], DSM-III- R [20], DSM- IV [21], DSM-IV-TR [22], and DSM-5 [23], or participants whose risk of suffering EDs was measured and for whom childhood trauma and the possible mediating factors were explored. The subjects included in the analysis were adolescents and adults. Additionally, these studies needed to be published in scientific journals, in either Spanish or English. Exclusion criteria were as follows: studies which did not explore the mediating factors existing between childhood trauma and the development of eating disorders, or which were published in a language other than English or Spanish. 

**Figure 1:**
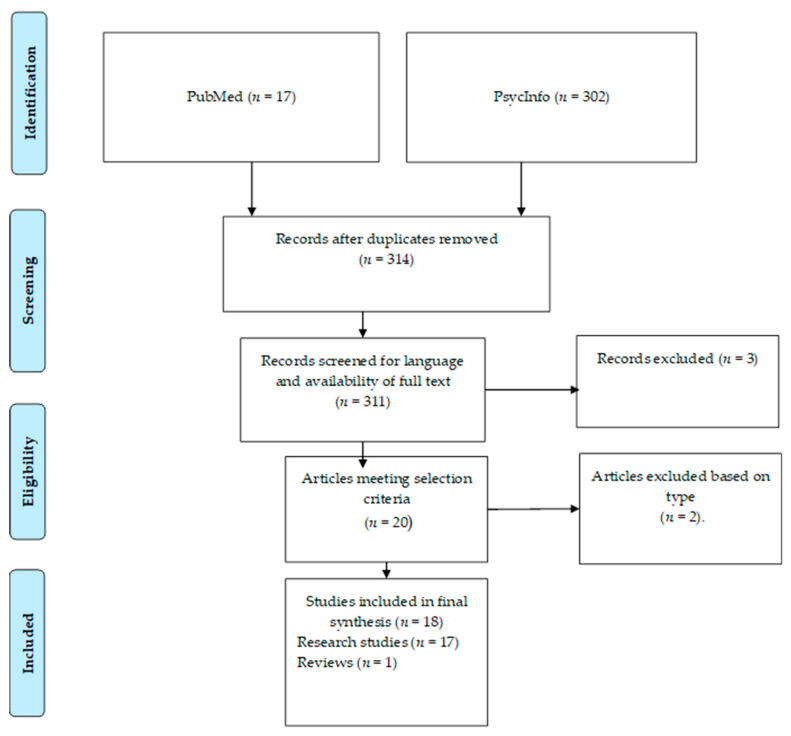
Flow chart indicating the steps taken in the systematic process of article selection.

A systematic review of the literature was carried out using the APA databases PsycInfo and PubMed up to 5 December, 2020. The search included studies published in any language. The keywords were “childhood trauma” AND “eating disorders” AND “mediating factors” and the combination between them. This search resulted in a total of 18 articles, from which the following data were extracted: names of the authors, year of publication, country of origin, characteristics of the participants, aim of the study, sample size, type of evaluation, and main results. The search process is shown in Figure 1.

A total of 319 articles were selected from both databases. After removing five duplicate articles, the rest were screened for language and availability of full text, of which a total of 311 articles met the criteria. We then proceeded to read all the articles to identify those which explored the mediating factors possibly existing between childhood traumatic experiences and the development of eating disorders, ultimately narrowing down the results to a total of 20 articles. Two more articles were then excluded based on type, for an end total of 18 studies to be included in the final synthesis.

## 3. Results

A total of 18 studies were retrieved after the eligibility criteria were applied: 17 research studies and one review. The list of selected research articles is shown in Table 1.

### 3.1. Participants

A total of 5738 subjects (the majority of them women) were included in the analysis. Of this total, 1269 subjects belonged to clinical populations whilst 4469 belonged to non-clinical populations.

### 3.2. Measures

To measure general eating pathology the instruments most used were the Eating Disorders Inventory (EDI-2) and Eating Attitudes Test (EAT-26) [15,24,27,28,29,31,35,36,37]. The EDI-2 evaluates eating disorder symptomatology and psychopathology. The questionnaire includes 11 subscales: ineffectiveness, social insecurity, drive to thinness, interoceptive awareness, maturity fear, body dissatisfaction, perfectionism, interpersonal distrust, impulsivity, bulimia, and ascetism. Cronbach’s values ranged from 0.75 (maturity fears) to 0.94 (ineffectiveness) in a recent study [36]. The EAT-26 is a 26-item scale used to assess eating disorder risk based on attitudes, feelings, and behaviors related to eating and eating disorder symptoms [35]. It consists of three subscales which are dieting, bulimia and food preoccupation, and oral control. Using a six-point Likert scale, they reported how often 26 statements about eating disorders attitudes and behaviors were true for them. The Eating Disorder Examination (EDE) was administered in a study too [9]. The EDE Global score was examined as an index of anorexia nervosa symptom severity over the past 28 days. The EDE Global score is calculated as the mean of four subscales (restraint, eating concern, shape concern, and weight concern). The psychometric properties of the EDE are well-established (α = 0.94–0.97) [9]. For specific aspects from EDs, The Bulimic Investigatory Test, Edinburgh (BITE) [28] for bulimia symptoms, Binge Eating Scale (BES) [34] for binge eating, and Disordered Eating Behaviors-Screening Questionnaire (DEB-SQ) for binge eating also [32,33] were used. The BITE test is a 33-item, self-report questionnaire that was designed as an objective screening test to identify subjects with bulimic symptoms. The BITE test consists of two subscales: the symptoms scale, and the severity scale. On the other hand, the BES is a 16-item self-report measure designed to assess for the presence and severity of binge eating symptoms [34]. Last, DEB-SQ is a self-report screening questionnaire which was developed for these studies [32,33]. It is a five-item Likert scale with 14 items. Half of the 14 items are consistent with the DSM-5 category of a binge eating disorder. The 7-item subscale of binge eating behaviors has an alpha reliability of 0.819 [32].

Overall, different instruments and methods to evaluate childhood trauma were used. Of all the studies, 55.6% used the same questionnaire to measure traumatic experiences, namely the Childhood Trauma Questionnaire-Short Form (CTQ-SF) [38]. The CTQ is an instrument for retrospective assessment of childhood maltreatment and neglect (emotional neglect, physical neglect, emotional abuse, physical abuse, and sexual abuse). It is a questionnaire with adequate psychometric properties [34]. Other instruments used to measure childhood traumatic experiences included the Child Abuse and Trauma Scale (CATS) [39] in the study by Kent et al. (1999) [24] and the study by Hund and Espelage (2005) [27]. The CATS is a 38-item questionnaire that assesses the frequency of various kinds of traumatic experiences suffered in childhood and adolescence, with adequate psychometric properties (the Cronbach’s alpha coefficient was 0.74) [27]. Other studies explored traumatic experiences through direct, dichotomous (yes/ no) questions asked during interviews [26,28,32,33], with questions like: As a child, do you remember being physically abused/sexually abused/verbally abused? [32]. Lastly, in the study by Malinauskiene and Malinauskas (2018) [35], childhood maltreatment was assessed using a 20-item retrospective questionnaire, the name of which was not specified.

In the case of dissociative symptomatology, all studies were using the Dissociative Experiences Scale (DES) [40]. The DES is a self-assessment questionnaire in which respondents must use percentages to indicate the frequency of 28 experiences, including difficulties involving memory, identity, absorption, depersonalization, and derealization. Each item is answered on a scale from 0 to 100. Scores beyond 30 are indicative of a dissociative disorder. This questionnaire is considered to have adequate psychometric properties [25]. With regard to assessing difficulties regulating emotions, the Difficulties in Emotion Regulation Scale (DERS) was used [41] in 66% of the studies measuring this variable [9,15]. The DERS scale has 36 items that assess difficulties regulating own emotions individually through adaptive regulation strategies. Each item receives a response on a frequency scale ranging from 1 (almost never) to 5 (almost always). The total score reflects the level of general difficulty regulating one’s own emotions. This questionnaire has adequate psychometric properties (internal consistency for the DERS total score was α = 0.93) [10]. The Regulation of Emotions Questionnaire (REQ) [42] was used in the study carried out by Mills et al. [31]. This questionnaire measures the use of functional and dysfunctional emotion regulation strategies using internal and external resources. It is specifically developed for adolescents, and its respondents must use a five-point scale to indicate the frequency with which a given strategy is used. It has a total of 21 items.

To measure levels of self-criticism, all studies incorporated the Rosenberg Self-Esteem Scale (RSES) [43]. This is a widely used self-esteem measure composed of 10 items. This scale has adequate internal consistency and excellent validity (α = 0.91) [11].

To measure anxiety and depressive symptomatology, different questionnaires were selected by the various authors: the Beck Depression Inventory (BDI) [44,45] to measure depressive symptomatology and the State-Trait Anxiety Inventory (STAI) to measure anxiety symptomatology [46]. The BDI is a 21-item questionnaire measuring symptomatology of depression. The STAI is a 40-item, self-report questionnaire that measures anxiety as a personal characteristic and as a state. This questionnaire has been shown to have adequate psychometric properties [27]. Both questionnaires have excellent internal consistency and validity with Cronbach’s alpha coefficient between 0.89 to 0.92 [11,34]. Other questionnaires used to measure this variable together with depressive symptomatology were the Hospital Anxiety and Depression Scale (HADS) [47] and the Brief Symptom Inventory (BSI) [48]. The HADS is a 14-item self-report questionnaire, composed of two subscales with seven items each: one for anxiety and the other for depression. The BSI measures anxiety and depression through 62 items with adequate psychometric properties (Cronbach’s alpha coefficient for the BSI depression and BSI anxiety subscales were 0.80 and 0.79, respectively) [32,33].

With respect to alexithymia, a consistent means of measuring this variable was used throughout the studies, with the Toronto Alexithymia Scale (TAS-20) [49] administered in both studies that measured this variable. TAS-20 is a self-report, 20-item scale evaluating difficulties in identifying and describing feelings, and has adequate psychometric properties (α = 0.83) [34].

Another variable that was explored as a mediator variable was body dissatisfaction, which was measured using the Body Attitudes Test (BAT) [50] and the Body Shape Questionnaire (BSQ) [51]. The BAT assesses individual subjective criteria about attitudes with respect to the body and attitudes towards it. The BSQ is made up of 34 items that measure the frequency with which concern over the size and shape of one’s body causes distress. Both questionnaires are considered to have adequate psychometric properties [11,28].

To measure post-traumatic stress symptoms, Malinauskiene and Malinauskas (2018) [35] used the Impact of Event Scale-Revised (IES-R) [52]. This scale is composed of 22 items measuring state of alert, anger, irritability, heightened startle response, difficulty concentrating, hypervigilance, and includes an intrusion subscale that measures flashbacks.

Core beliefs were analyzed by Waller et al. (2001) [26] using the Young Schema Questionnaire (YSQ) [53]. The YSQ is a self-report questionnaire made up of 205 items with subscales that measure 16 core beliefs.

Finally, to measure inefficiency and interoceptive awareness as possible mediating factors, the Eating Disorder Inventory (EDI-2) was used in the study by Monteleone et al. (2019) [36], and food addiction as a mediating factor was measured using the Yale Food Addiction Scale 2. 0 (YFAS 2.0) in the study by Khalil et al. (2020) [37].

### 3.3. Findings of the Studies of Non-Clinical Populations

All the studies analyzed employed a cross-sectional design [15,24,27,28,31,32,33,34,35].

The studies carried out within non-clinical populations make up 56.62% of the total sample—both among students [15,24,27,31,35] and the general population [28,32,33,34]. Ten percent of the participants were adolescents [31], 90% adults [15,24,25,27,28,32,33,34,35]. 

In the Moulton study [15], in addition to dissociation as a mediating factor, difficulty regulating emotions was explored as another possible mediator variable through a multiple mediation model, which concluded that both dissociation and difficulty regulating emotions are significant mediators between childhood trauma and EDs [15]. These results are similar to the findings by Mills et al. (2015) [31], whose purpose was to determine whether regulation of emotions mediates the relationship between emotional abuse and EDs in a sample of adolescents. The results of this study showed that EDs are associated with emotional abuse, emotion dysregulation, and being female. The multiple mediation analysis revealed an indirect link between emotional abuse and EDs via emotion dysregulation.

The work of Kent et al. (1999) [24] explored anxiety and depression as possible mediators, in addition to dissociation. The findings concluded that emotional abuse was the only kind of childhood trauma that predicts EDs in adulthood and that this relationship is mediated in women by levels of anxiety and dissociation. However, a later study exploring anxiety, depression, anger, and self-criticism as possible mediator variables concluded that anger and self-criticism did mediate significantly between childhood trauma and EDs, but that anxiety and depression did not [33]. The same authors [32] found similar results in another study on the role of self-criticism, anxiety, and depression as mediators between emotional abuse during childhood and binge eating disorders. Other possible mediators explored in other studies have included the presence of symptoms of post-traumatic stress [35], body dissatisfaction [28], alexithymia [27,34], and general distress [27].

Recently, Malinauskiene and Malinauskas (2018) [35] studied the role of symptoms of post-traumatic stress, confirming the mediating role between trauma and EDs. However, in this study it is important to remark that the trauma type included were accidents in which individuals were severely injured and they did not always occur in childhood. Body dissatisfaction, due to its relationship with EDs and sexual abuse, has also been studied as a possible mediating factor between sexual abuse and EDs, its role being confirmed in the study by Preti et al. (2006) [28].

As for alexithymia, various studies confirm it can function as a mediator between childhood traumatic experiences and EDs [27,34]. One study explored the relationship between physical and emotional neglect in childhood and binge eating disorders and muscle dysmorphia in a sample of 1344 individuals, including men and women [34]. The study confirms that the relationship between emotional and physical neglect in childhood and symptoms related to binge eating disorders are mediated by alexithymia. The study by Hund and Espelage (2005) [27], in addition to alexithymia, explored general distress as a mediator and concluded that it also mediated the relationship between variables.

### 3.4. Findings in the Studies of Clinical Populations

Again, all studies were of a cross-sectional design in nature [9,11,25,26,29,30,36,37], with one exception of one review study [17].

The studies carried out on clinical populations make up 44.4% of the total sample. The subjects were receiving various psychiatric diagnoses: binge eating disorder [11,25,26,37], anorexia nervosa [9,25,26,30,36,37], adolescents with multiple pathologies that include EDs [36], bulimia [25,26,29,37], and other specified feeding or EDs [29]. Overall, 42.8% were adolescents [9,29,30], while 57.1% were adults [9,25,26,29,36,37].

Self-criticism [11] and dissociation [26] were explored as mediators between childhood traumatic experiences and binge eating disorders. Findings show that self-criticism is a potential mediator through which some forms of abuse are associated with depressive symptomatology and body dissatisfaction in individuals with binge eating disorders. The study by Waller et al. (2001) [28] studies the presence of dissociation in an ED sample, based on an understanding of the dissociation as a dimensional construct that varies depending on the ED subtype, chiefly those which are impulsive and are associated with greater ED severity.

In individuals with anorexia nervosa, the mediating factors studied included emotion regulation [9], depression [29], obsession-compulsion [29], interoceptive awareness [36], inefficiency [36], food addiction [37], and dissociation [25]. With respect to emotion regulation as a possible mediator, emotional abuse was the type of abuse that was most related to difficulties with emotion regulation and with the severity of symptoms of anorexia nervosa. The lack of emotion regulation significantly explained the relationship between emotional abuse in childhood and the symptoms associated with anorexia nervosa [9]. In another study carried out in South Korea aiming at determining the specific forms of childhood trauma that may predict a future development of an eating disorder, the presence of both depressive and obsessive–compulsive symptoms were explored as mediating factors [29]. Emotional abuse, physical neglect, and sexual abuse were the most significant predictors of EDs. The study also concluded that depressive symptomatology mediates the relationship between childhood trauma and EDs, whilst obsessive-compulsive symptoms did not. Differences by age groups were not explored in this study for these variables [29]. Another study focusing on interoceptive awareness and inefficiency concluded that these variables did mediate the possible association between childhood trauma and the development of anorexia nervosa [36].

In the studies of dissociation as a possible mediating factor in anorexia nervosa [25,26], the results indicate that individuals with EDs show symptoms of dissociation especially in eating disorder patients of the purging type. Hopwood et al. (2011) [30] explored the mediating role of depressive/negative affectivity in multiple types of childhood maltreatment, in addition to ED characteristics in a sample of adolescents hospitalized in a general psychiatric unit who had received various psychiatric diagnoses, including ED diagnoses. The results of this study suggested that, in the case of the girls, depressive/negative affectivity significantly mediated the relationship between childhood trauma and the EDs, although the effects varied depending on the type of abuse and the specific ED traits. Generalization of mediation effects to boys was limited.

Other researchers focused on core beliefs (maladaptive early beliefs) as potential mediators between sexual abuse in women with bulimia nervosa [26]. Of the 61 individuals who made up the sample, 21 reported having suffered sexual abuse in childhood. Individuals reporting sexual abuse, as compared to the non-abused sample, reported a greater number of maladaptive core beliefs and significantly more psychopathology. Different maladaptive core beliefs acted as mediators in the relationships between sexual abuse and individual symptoms in this sample. The authors concluded [26] that different maladaptive core beliefs act as mediators in the relationship between sexual abuse and symptoms.

Finally, a recent study [37] concludes that certain kinds of childhood maltreatment induce, maintain, or worsen the presence of EDs through the mediating effect of food addiction as a possible variable.

## 4. Discussion

Based on these findings, the results show that various variables may function as a possible mediator between childhood trauma and the development of an ED, such as anxiety, dissociation, core beliefs, depressive symptomatology, alexithymia, body dissatisfaction, self-criticism, difficulty with emotion regulation, anger, inefficiency, interoceptive awareness, food addiction, and symptoms of post-traumatic stress. This is a wide variety with respect to the number of articles reviewed. The variables most studied as possible mediators were depressive symptomatology, anxiety, and dissociation.

With regard to the possible mediating role of dissociation, the results clearly show that dissociation functions as a mediator between childhood traumatic experiences and EDs. In the studies carried out in non-clinical populations, dissociation seems to work as a mediating factor between having suffered childhood trauma and later development of an ED [15,24]. This finding corroborates results of other studies in which dissociation is understood as a defense mechanism allowing an individual to escape from the distress caused by unresolved trauma [12,13]. Similarly, in clinical populations, Waller et al. (2001) [26] postulated that dissociation serves as a means of escape from conscious awareness of unbearable emotions and cognitive states, manifested mostly in impulsive ED subtypes and being associated with more severe symptoms.

The variables anxiety and depressive symptomatology were also frequently explored; however, their possible mediating role remains somehow unclear [32,33]. In some studies, depression mediates the relationship between some forms of traumatic experiences in childhood and EDs significantly [29,30]. However, in others, anxiety and depressive symptomatology appear as covariates in relation to self-criticism and not as direct mediators [32,33]. Anxiety and depressive symptoms can be symptoms that develop due to not having resolved childhood traumatic experiences and may be associated with difficulty regulating emotions. Therefore, its early approach can facilitate the regulation of patients adaptively instead of regulating their emotions through an ED.

The results regarding the mediating role of body dissatisfaction and alexithymia remain in line with previous studies [8,14] for those reviewed in non-clinical populations [27,28,34]. Emotional and physical neglect and sexual abuse are associated with the development of EDs, and this relationship seems to be mediated by alexithymia. Alexithymia can function in a similar way to dissociative symptomatology, allowing patients not to feel what they cannot tolerate in relation to unresolved childhood trauma. Additionally, the impact of sexual abuse on the development of EDs seems to be mediated by the presence of body dissatisfaction [28]. Body dissatisfaction in experiences of sexual abuse can appear as a form of rejection of the experience of abuse itself. The mediating role of self-criticism is associated with the severity of binge eating as both a mediator [32] and an associated variable [32,43], as is reflected in the findings of the study carried out by Everill and Waller (1995) [10].

### 4.1. Limitations

Several limitations need to be mentioned. Firstly, the heterogeneity of the participants included in this review, both clinical and non-clinical samples, is a limiting factor. Next, consistency is lacking in the instruments used to measure the different variables. Different instruments, methods, and questionnaires were used, and the majority of the studies did not evaluate the subjective impact and/or severity of the traumatic experiences suffered. These are limitations which have already been highlighted in previous studies [15,16] but limit the generalizability of our findings.

### 4.2. Future Research Directions

Future studies are recommended to further explore these mediators between childhood traumatic experiences and EDs. It is important because childhood traumatic variables were found to explain only a small part of EDs. Future research also should consider whether patients with a history of childhood traumatic experiences may benefit most from treatments for EDs that aim to target the mediator variables assessed in each clinical case.

### 4.3. Implications in clinical practice

The results underline the importance to systematically assess the presence of traumatic experiences in eating disorder patients together with a wide range of possible mediator variables that may have an impact on the future development of the eating disorder symptoms. These childhood traumatic experiences are, in many cases, associated with the severity of the symptoms being suffered by the patient and the motive for which therapeutic treatment has been started. EDs are often preceded by experiences that have caused profound harm to people [54].

When childhood trauma is present, the eating disorder may have a different function compared to the classical eating disorder cases, such as being afraid to have a normal body weight. In the case of trauma, the eating disorder may function as a survival mechanism, and may have a protective function to avoid the emotional confrontation with the trauma experience. It is important that the therapist is aware of this possibility and tries to understand the meaning of the eating disorder as such. Therefore, the normalization of weight, for instance, in the case of anorexia nervosa, needs to be carefully planned since it may provoke a strong confrontation with the memories and emotions related to the traumatic past. Trauma stabilization techniques must be introduced in the treatment of trauma patients together with therapeutic strategies to work through the trauma experiences, once some symptom stabilization has been reached. 

In conclusion, it is important to systematically assess the presence of trauma and to take into account these mediating factors. In case trauma is present, we suggest introducing a trauma-informed approach and to detect and/or evaluate possible symptoms or risk factors that could predict a later development of complex EDs. Child health care providers should consider an early approach to these traumatic experiences suffered in childhood, as it could prevent the development of more severe disorders in adulthood.

## Figures and Tables

**Table 1:** List of selected research articles.

First Author and Years of Publication	Country	Sample	ED Diagnosis in Clinical Population Studies	Mediators	Questionnaires Used to Assess Mediators
Kent, 1999 [24]	UK	236 adults NCS	-	Anxiety and dissociation	DES and HADS
Waller, 2001 [25]	UK	170 adults CS and 203 adults NCS	Bulimia nervosa, anorexia nervosa, and binge eating disorder	Dissociation	DES II
Waller 2001 [26]		61 adults CS	Bulimia nervosa, anorexia nervosa, and binge eating disorder	Dissociation, core beliefs, and depressive symptomatology	DES II, YSQ, and BDI
Hund, 2005 [27]	USA	608 adults NCS	-	General distress and alexithymia	STAI and TAS-20
Preti, 2006 [28]	Italy	126 adults NCS	-	Body dissatisfaction	BAT
Kong, 2009 [29]	South Korea	73 adolescents and adults CS	Anorexia nervosa, bulimia nervosa, and eating disorder not otherwise specified	Depressive symptomatology	BDI
Dunkley, 2010 [11]	USA	170 adults CS	Binge eating disorder	Self-criticism and body dissatisfaction	RSES and BSQ
Hopwood, 2011 [30]	USA	148 adolescents CS	Not specified but including EDs	Depressive symptomatology	BDI
Mills, 2015 [31]	UK	222 adolescents NCS	-	Difficulties regulating emotions	REQ
Moulton, 2015 [15]	UK	142 adults NCS	-	Difficulties regulating emotions and dissociation	DERS and DES II
Racine, 2015 [9]	USA	188 adolescent and adults CS	Anorexia nervosa	Difficulties regulating emotions	DERS
Feinson, 2016 [32]	Israel	498 adults NCS	-	Self-criticism, anxiety, and depressive symptomatology	RSES and BSQ
Feinson, 2016 [33]	Israel	476 adults NCS	-	Self-criticism, anger, anxiety, and depressive symptomatology	RSES and BSQ
Minnich, 2017 [34]	USA	1344 adults NCS		Alexithymia	TAS-20
Malinauskiene, 2018 [35]	Lithuania	614 adults NCS		Symptoms of post-traumatic stress	IES-R
Monteleone, 2019 [36]	Italy	228 adults CS	Anorexia nervosa restricting type and anorexia nervosa binge-purging type	Interoceptive awareness and inefficiency	STAI and EDI-2
Khalil, 2020 [37]	France	231 adults CS	Anorexia nervosa, bulimia nervosa, binge eating disorder, and other types of eating disorders	Food addiction	YFAS 2.0

Note. ED = eating disorders, CS = clinical sample, NCS = non-clinical sample, HADS = Hospital Anxiety and Depression Scale, YSQ = Young Schema Questionnaire, STAI = State-Trait Anxiety Inventory, DES = Dissociative Experiences Scale, TAS-20 = Toronto Alexithymia Scale, BAT = Body Attitudes Test, BDI = Beck Depression Inventory, RSES = Rosenberg Self-Esteem Scale, BSQ = Body Shape Questionnaire, REQ = Regulation of Emotions Questionnaire, DERS = Difficulties in Emotion Regulation Scale, IES-R = Impact of Event Scale-Revised, EDI-2 = Eating Disorders Inventory, YFAS 2.0 = Yale Food Addiction Scale 2.0.
